# Porto-Sinusoidal Vascular Liver Disease Associated With Trastuzumab Emtansine: A Case Report

**DOI:** 10.7759/cureus.85981

**Published:** 2025-06-14

**Authors:** Ana Rita Antunes, Francisca Carmo, Adriana Pereira Guedes, Catarina Antunes Salvado, Isabel O Cruz

**Affiliations:** 1 Internal Medicine, Unidade Local de Saúde de Gaia e Espinho, Vila Nova de Gaia, PRT; 2 Medical Sciences, University of Aveiro, Aveiro, PRT

**Keywords:** drug-induced vascular liver disease, elevated liver enzymes, her2-positive invasive breast cancer, porto-sinusoidal vascular disorder, trastuzumab emtansine

## Abstract

Porto-sinusoidal vascular disorder (PSVD) is a rare vascular liver disease characterised by alterations in the portal and sinusoidal vasculature in the absence of cirrhosis, often associated with immune conditions, haematological disorders, or medication exposure. We describe a 69-year-old woman with a history of human epidermal growth factor receptor 2 (HER2)-positive invasive breast cancer, treated with 15 cycles of trastuzumab emtansine (T-DM1) from January to October 2020. From the third cycle, she developed an increase in aminotransferase levels to three times the upper limit of normal, which normalized after discontinuation of T-DM1 due to cancer progression. Two years later, she presented to the emergency department with hepatic encephalopathy, lower limb oedema, and signs of portal hypertension. Laboratory tests showed thrombocytopenia and a cytocholestatic pattern, and hepatic venous pressure gradient confirmed portal hypertension. Liver biopsy revealed histological features consistent with PSVD without cirrhosis. A diagnosis of drug-induced PSVD was made, likely secondary to T-DM1. The patient was treated with diuretics and lactulose, with marked clinical improvement and no further complications over one year of follow-up. This case underscores the importance of considering PSVD in patients with unexplained liver dysfunction and portal hypertension following cancer therapy.

## Introduction

Porto-sinusoidal vascular disorder (PSVD) is a rare cause of portal hypertension and encompasses a group of vascular liver conditions characterised by specific alterations in the portal and sinusoidal vasculature [1-3]. Previously categorised under non-cirrhotic portal hypertension (NCPH), PSVD is distinguished by a combination of clinical and histopathologic findings in the absence of cirrhosis, including nodular regenerative hyperplasia, obliterative portal venopathy, portal vein stenosis, and incomplete septal fibrosis [1-4]. These vascular changes may occur with or without clinically evident portal hypertension and are typically confirmed through liver biopsy, which remains the gold standard for diagnosis [1-4].

PSVD is frequently associated with underlying conditions such as immunological or haematological disorders, as well as exposure to certain medications [2]. Prognosis tends to be less favourable in patients with more severe underlying disease and is further worsened when ascites is the initial presentation, or in the presence of elevated bilirubin and creatinine levels, and low serum albumin concentrations [2].

Trastuzumab emtansine (T-DM1) is an antibody-drug conjugate consisting of trastuzumab, a monoclonal antibody directed against human epidermal growth factor receptor 2 (HER2), linked to emtansine, an antimicrotubule agent [4,5]. It is approved for third-line or later treatment for patients with HER2-positive, locally advanced, unresectable, or metastatic breast cancer who have previously received trastuzumab and a taxane, either as monotherapy or in combination therapy [5,6]. Although T-DM1 has demonstrated a favourable safety profile and improved progression-free and overall survival, recent evidence suggests a potential association with PSVD [5,6]. The most frequently reported grade 3 or 4 adverse events include thrombocytopenia and elevated serum levels of aspartate aminotransferase (AST) and alanine aminotransferase (ALT) [7,8]. The emtansine (DM1) component may cause direct endothelial injury by disrupting microtubule dynamics in endothelial cells, resulting in vascular dysfunction. Additionally, trastuzumab has been linked to immunologic mechanisms that may promote endothelial activation or injury, particularly in susceptible individuals. These effects could contribute to portal venopathy and other vascular changes seen in rare cases of PSVD [9].

This case highlights an uncommon presentation of portal hypertension in the setting of porto-sinusoidal vascular liver disease, potentially following treatment with T-DM1, underscoring the need for awareness of this association in clinical practice.

## Case presentation

**Figure 1:**
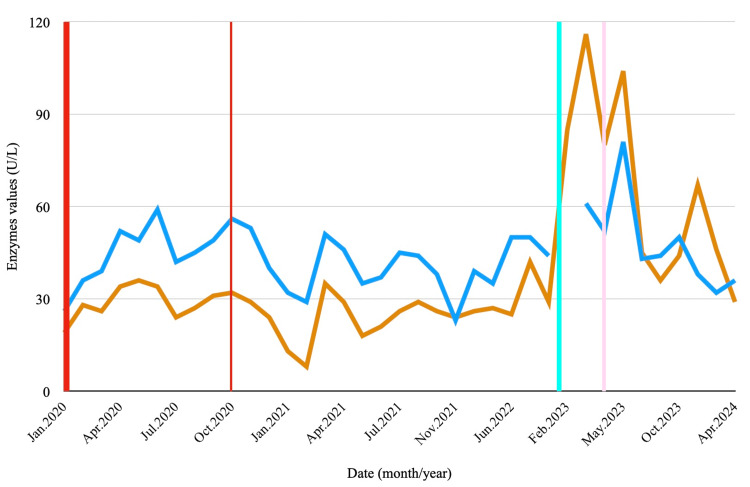
Evolution of aspartate aminotransferase and alanine aminotransferase, reflecting potential liver dysfunction and treatment effects. The orange line represents the progression of alanine aminotransferase (ALT) levels, and the dark blue line represents aspartate aminotransferase (AST) levels. The bold red line marks the start of treatment with trastuzumab emtansine (T-DM1), while the thin red line indicates the end of treatment. The light blue line denotes the asymptomatic elevation of liver enzymes at the follow-up visit, and the light pink line indicates hospitalisation due to worsening liver disease. The x-axis shows the date (month/year), and the y-axis indicates enzyme levels in units per litre (U/L).

We report a case of a 69-year-old British woman, living in Portugal since 2019, diagnosed in 2014 with HER-2-positive invasive carcinoma of the right breast and submitted to multiple treatments due to local relapse and cutaneous metastasis. Of note, the patient was treated with 15 cycles of TDM-1 from January to October of 2020, and serum aminotransferase levels rose to three times the upper limit of normal (ULN) from the third cycle on, but normalised after drug suspension, prompted by disease progression (Figure 1). Due to further progression of cutaneous lesions, treatment was altered to capecitabine and lapatinib and then trastuzumab deruxtecan, until January 2023. The latter was discontinued after the development of grade 3 interstitial lung disease, for which the patient was started on corticosteroid therapy.

**Figure 2:**
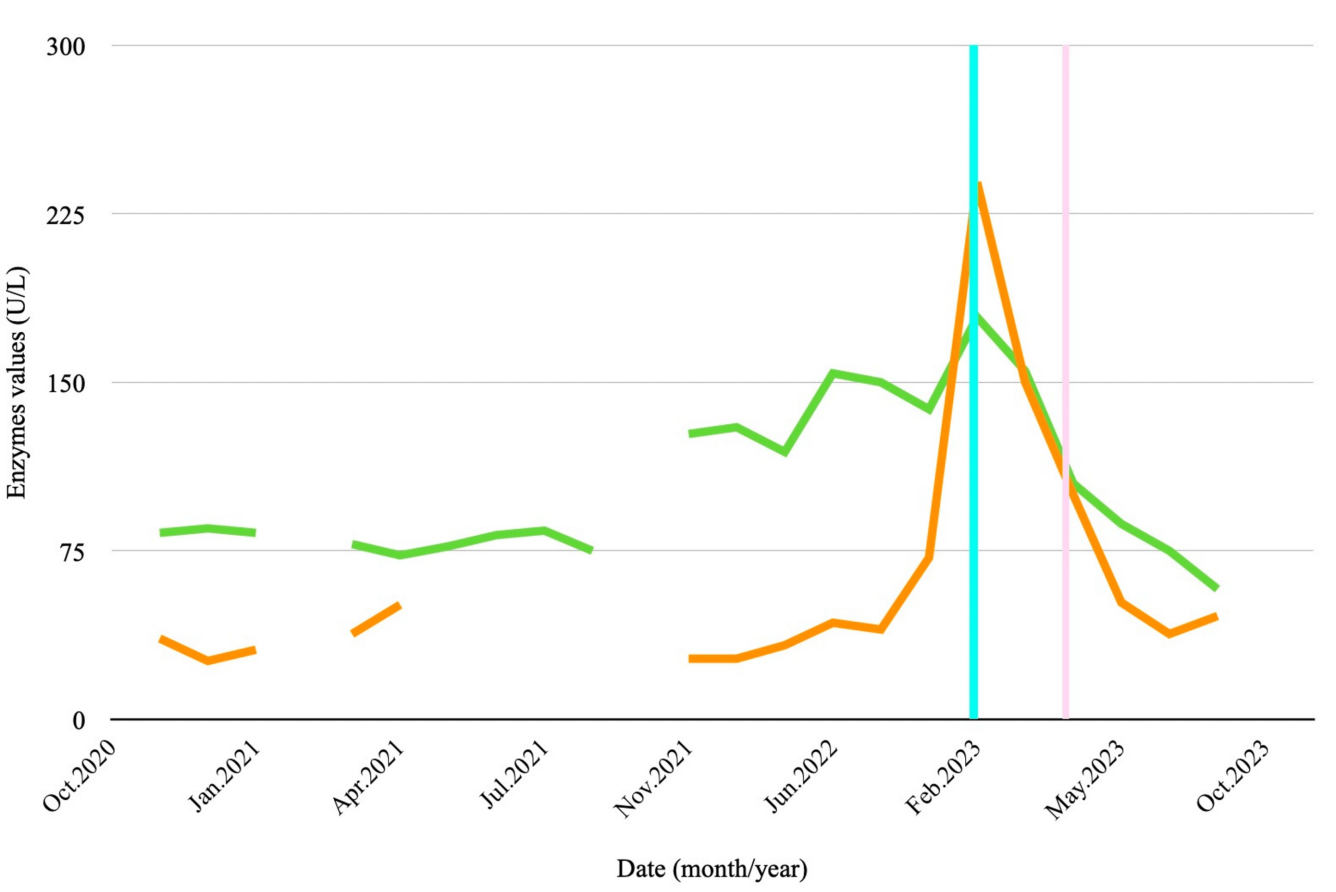
Evolution of alkaline phosphatase and gamma-glutamyl transpeptidase, reflecting potential liver dysfunction and treatment effects. The light orange line represents the progression of gamma-glutamyl transpeptidase (GGT) levels, and the green line represents alkaline phosphatase (ALP) levels. The light blue line denotes the asymptomatic elevation of liver enzymes observed at the follow-up visit, while the light pink line indicates hospitalisation due to worsening liver disease. The x-axis shows the date (month/year), and the y-axis indicates enzyme levels in units per litre (U/L).

**Figure 3:**
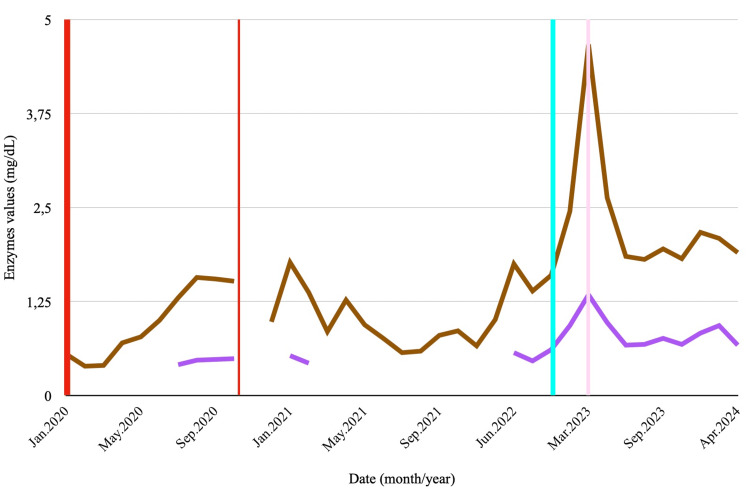
Evolution of total bilirubin and direct bilirubin, reflecting potential liver dysfunction and treatment effects. The brown line represents the progression of total bilirubin (TB) levels, and the purple line represents indirect bilirubin (ID) levels. The bold red line marks the start of treatment with trastuzumab emtansine (T-DM1), while the thin red line indicates the end of treatment. The light blue line denotes the asymptomatic elevation of liver enzymes observed at the follow-up visit, and the light pink line indicates hospitalisation due to worsening liver disease. The x-axis shows the date (month/year), and the y-axis indicates enzyme levels in units per litre (U/L).

In February 2023, routine labs showed elevated liver enzymes, gamma-glutamyl transpeptidase (GGT), an increase in total bilirubin (TB) mainly due to indirect bilirubin (ID), and newly developed thrombocytopenia (Figures 1-3). There was no introduction of new potentially hepatotoxic medications, and imaging studies revealed no hepatic lesions, biliary tract dilatation, ascites, or signs of portal hypertension.

In March 2023, she was hospitalised in a British hospital for an acute confusional state of undetermined aetiology. Infections and brain metastasis were excluded. She recovered spontaneously and was discharged. Later that month, the patient remained asymptomatic, but follow-up blood work revealed elevations in all liver enzymes (Figures 1-3).

In April 2023, the patient presented to the emergency department with somnolence, dyspnoea on minimal exertion, orthopnoea, paroxysmal nocturnal dyspnoea, and progressive lower limb oedema over the preceding week. She denied any other symptoms. On examination, she was lethargic but responsive, with lower limb oedema extending up to the groin, jugular venous distension, and bibasilar crackles. There was no asterixis or disorientation. Laboratory tests on admission revealed thrombocytopenia and a cytocholestatic pattern, with elevated alkaline phosphatase (ALP), GGT, TB, direct bilirubin (DB), AST, and ALT. Urinalysis was unremarkable. Diuretic therapy was initiated, and she was admitted to the internal medicine department for stabilisation and further evaluation.

During hospitalisation, a cardiac cause was initially considered given the clinical signs of volume overload and the patient’s favourable response to diuretics; however, normal N-terminal pro-B-type natriuretic peptide (NT-proBNP) levels and an unremarkable transthoracic echocardiogram were not consistent with a cardiac origin. Renal causes were also explored, but although hypoalbuminaemia was present, there was no accompanying proteinuria. Capillary leak syndrome was considered unlikely, as it typically presents with a more acute clinical course, responds to fluid therapy rather than diuretics, and does not match the patient’s disease progression. Abdominal CT scan revealed a small volume of ascites, with no evidence of focal liver lesions, bile duct dilatation, splenomegaly, or thrombosis in the splanchnic circulation. Hepatic venous pressure gradient (HVPG) measurement confirmed clinically significant portal hypertension, with a value of 18 mmHg.

Liver biopsy provided an adequate tissue sample (20 mm in length with 28 portal tracts). Histology showed mild to moderate portal inflammation, obliterative portal venopathy with portal vein stenosis, sinusoidal obstruction with lobular congestion, and incomplete septal fibrosis in periportal areas, without evidence of cirrhosis. No malignant cell infiltration was identified. Thyroid function tests were within normal limits. Although the patient’s morning cortisol level was low (1.8 μg/dL; normal range: 6.2-19.4 μg/dL), this was attributed to chronic prednisolone use and was not considered clinically significant. Cerebral CT ruled out brain metastases.

Based on these findings, the patient fulfilled the diagnostic criteria for PSVD, with thrombocytopenia and a liver biopsy showing characteristic histological features in the absence of cirrhosis. Upper endoscopy excluded the presence of oesophageal varices. T-DM1 was identified as the most likely causative agent, while immunological and haematological causes were excluded.

Treatment with intravenous furosemide and oral spironolactone led to rapid clinical improvement and achievement of euvolaemia within days. Lactulose was added, and two to three daily bowel movements resulted in the resolution of neurological symptoms. Both medications were continued at discharge, and the patient was referred for hepatology follow-up. She has since remained stable without further complications and has not resumed T-DM1 therapy.

At one-year follow-up, the patient demonstrated sustained clinical improvement, with liver function tests showing progressive stabilisation.

## Discussion

PSVD is an uncommon and frequently under-recognised condition, primarily due to the absence of pathognomonic clinical, biochemical, radiological, or histopathological features [1-3]. As a result, diagnosis is often delayed, and PSVD may be misinterpreted as cirrhosis or other causes of portal hypertension [1-3].

Recent literature has led to increased awareness of PSVD and a broader understanding of its heterogeneous clinical presentations [2]. Most patients are asymptomatic at the time of diagnosis, with incidental findings such as thrombocytopenia, elevated liver enzymes, or imaging abnormalities, particularly splenomegaly [2]. Among symptomatic individuals, ascites is the most frequently reported manifestation, followed by variceal bleeding, a combination of both, dyspnoea, and hepatic encephalopathy [2]. The presence and severity of associated disorders also play a critical role in raising clinical suspicion for PSVD and are associated with poorer prognosis [1-4].

In patients with underlying conditions presenting with such findings, regardless of symptomatology, PSVD should be considered in the differential diagnosis. Liver biopsy remains essential to exclude cirrhosis and to identify specific or non-specific histological lesions suggestive of PSVD [1-5].

In this case, our patient developed elevated aminotransferase levels following the third cycle of T-DM1 in March 2020. These abnormalities normalised within two to three months after discontinuation of the drug and remained stable during follow-up. The absence of alternative causes, coupled with the temporal association between the initiation of T-DM1 and the onset of liver enzyme abnormalities, and their resolution following cessation, strongly supports the role of T-DM1 in triggering PSVD, a relationship that has been described in the literature [10-13].

In February 2023, despite no introduction of new hepatotoxic agents and normal imaging studies, the patient developed thrombocytopenia, elevated aminotransferases, raised GGT, and increased TB. In March, she was admitted with an acute confusional state of unclear aetiology, followed by further elevation of hepatic enzymes.

By April 2023, she presented with hepatic encephalopathy, decompensated oedematous-ascitic syndrome, thrombocytopenia, and a cytocholestatic pattern. Taking into account the past medical history, clinical course, biochemical progression, and histological findings, namely, obliterative portal venopathy, mild to moderate portal inflammation, and incomplete septal fibrosis without cirrhosis, the diagnosis of PSVD was confirmed. Although liver enzyme levels improved after T-DM1 discontinuation, the vascular damage appears to have led to persistent susceptibility, ultimately resulting in delayed hepatic decompensation.

In this patient, the absence of cirrhosis on liver biopsy, together with one specific histological feature of PSVD, obliterative portal venopathy with incomplete septal fibrosis, fulfilled the diagnostic criteria for PSVD. Additionally, she exhibited two non-specific signs of portal hypertension, ascites and thrombocytopenia, along with one non-specific histological feature, sinusoidal dilatation [1]. Given the temporal relationship and absence of other potential causes, T-DM1 was considered the most likely trigger.

The primary therapeutic measure in drug-induced PSVD is discontinuation of the causative agent, which had already been implemented in this case [12,13]. The patient’s anasarca was successfully managed with a combination of furosemide and spironolactone, while lactulose proved essential for the treatment and prevention of constipation and hepatic encephalopathy. This therapeutic strategy aligns with current approaches to managing portal hypertension, with a focus on symptom control and the prevention of further complications.

## Conclusions

This case highlights PSVD as a potential, albeit rare, complication of T-DM1 therapy. PSVD should be considered in the differential diagnosis of patients undergoing systemic cancer treatment or with relevant associated conditions who present with thrombocytopenia, unexplained liver dysfunction, and clinical or laboratory features of portal hypertension in the absence of cirrhosis. Liver biopsy remains essential for definitive diagnosis, as non-invasive imaging may not detect the subtle vascular lesions characteristic of PSVD. Management requires prompt discontinuation of the offending agent, followed by supportive treatment focused on controlling portal hypertension and preventing further complications. In this patient, such an approach resulted in sustained clinical improvement and progressive stabilisation of liver function tests at one-year follow-up.

This case reinforces the importance of maintaining a high index of suspicion, enabling early recognition, timely withdrawal of the causative therapy, and appropriate clinical management to optimise patient outcomes.
